# Dopamine-dependent, swimming-induced paralysis arises as a consequence of loss of function mutations in the RUNX transcription factor RNT-1

**DOI:** 10.1371/journal.pone.0216417

**Published:** 2019-05-13

**Authors:** Sarah B. Robinson, Osama Refai, J. Andrew Hardaway, Sarah Sturgeon, Tessa Popay, Daniel P. Bermingham, Phyllis Freeman, Jane Wright, Randy D. Blakely

**Affiliations:** 1 Department of Pharmacology, Vanderbilt University Medical Center, Nashville, TN, United States of America; 2 Department of Biomedical Science, Charles E. Schmidt College of Science, Florida Atlantic University, Jupiter, FL United States of America; 3 Department of Life and Physical Sciences, Fisk University, Nashville, TN, United States of America; 4 Brain Institute, Florida Atlantic University, Jupiter, FL, United States of America; CSIR-Central Drug Research Institute, INDIA

## Abstract

Dopamine (DA) is a neurotransmitter with actions across phylogeny that modulate core behaviors such as motor activity, reward, attention, and cognition. Perturbed DA signaling in humans is associated with multiple disorders, including addiction, ADHD, schizophrenia, and Parkinson’s disease. The presynaptic DA transporter exerts powerful control on DA signaling by efficient clearance of the neurotransmitter following release. As in vertebrates, *Caenorhabditis elegans* DAT (DAT-1) constrains DA signaling and loss of function mutations in the *dat-1* gene result in slowed crawling on solid media and swimming-induced paralysis (Swip) in water. Previously, we identified a mutant line, *vt34*, that exhibits robust DA-dependent Swip. *vt34* exhibits biochemical and behavioral phenotypes consistent with reduced DAT-1 function though *vt34; dat-1* double mutants exhibit an enhanced Swip phenotype, suggesting contributions of the *vt34*-associated mutation to additional mechanisms that lead to excess DA signaling. SNP mapping and whole genome sequencing of *vt34* identified the site of the molecular lesion in the gene B0412.2 that encodes the Runx transcription factor ortholog RNT-1. Unlike *dat-1* animals, but similar to other loss of function *rnt-1* mutants, *vt34* exhibits altered male tail morphology and reduced body size. Deletion mutations in both *rnt-1* and the *bro-1* gene, which encodes a RNT-1 binding partner also exhibit Swip. Both *vt34* and *rnt-1* mutations exhibit reduced levels of *dat-1* mRNA as well as the tyrosine hydroxylase ortholog *cat-2*. Although reporter studies indicate that *rnt-1* is expressed in DA neurons, its re-expression in DA neurons of *vt34* animals fails to fully rescue Swip. Moreover, as shown for *vt34*, *rnt-1* mutation exhibits additivity with *dat-1* in generating Swip, as do *rnt-1* and *bro-1* mutations, and *vt34* exhibits altered capacity for acetylcholine signaling at the neuromuscular junction. Together, these findings identify a novel role for *rnt-1* in limiting DA neurotransmission and suggest that loss of RNT-1 may disrupt function of both DA neurons and body wall muscle to drive Swip.

## Introduction

Dopamine (DA) is a critical modulator of brain circuits that drive fundamental behaviors such as movement, habit, reward, attention, and cognition and thus, not surprisingly, DA signaling alterations are evident in human motor disorders, such as Parkinson’s disease and dystonia, in the actions of substances of abuse [1, 2], and in disorders that feature a disruption of higher brain function, including attention-deficit/hyperactivity disorder (ADHD) [3, 4], bipolar disorder [5–7] and schizophrenia [8, 9]. The complexities and limited accessibility of the human brain has led many investigators to adopt simpler model systems to dissect DA signaling, including the fruitfly *Drosophila melanogaster* [10–12] and, in our case, the nematode *Caenorhabditis elegans [13]*. *C*. *elegans* DA signaling has proven suitable for both pharmacological and genetic studies of human DA modulatory genes [14–16] owing to the conservation of genes that encode DA synthesis, vesicular packaging, response, and inactivation [17].

The current report originates from our efforts to define novel, conserved determinants of DA signaling via a forward genetic approach [18, 19], whereby mutant animals are identified based on their exhibition of a readily detectible phenotype arising in the context of excess DA signaling, termed swimming-induced paralysis [20]. We first identified Swip in animals possessing loss of function alleles in the *dat-1* gene that encodes the presynaptic DA transporter (DAT-1) [20]. As with mammalian orthologs, synaptic clearance of DA by DAT-1 limits the actions of DA in time and space on pre- and postsynaptic DA receptors. An inability to clear synaptic DA, in the context of significant DA release triggered by immersion of worms in water, leads to extrasynaptic actions of DA on the D2-type DA receptor DOP-3 that inhibit the activity of motorneurons, leading to paralysis.

Swip induced by *dat-1* mutations can be prevented by growth of worms on reserpine, an inhibitor of the vesicular monoamine transporter (VMAT/CAT-1) [21], and pharmacologically reversed by treatment of *dat-1* paralyzed worms by the DOP-3 antagonist azaperone [22]. To identify novel loss of function alleles in *dat-1*, as well as novel determinants of DA signaling, we undertook a forward genetic screen [18] that identified lines displaying reserpine-sensitive Swip, as well as Swip sensitivity to DOP-3 genetic elimination or azaperone treatment. Here we present characterization of a mutant line identified in this screen, *vt34*. We identify the molecular lesion in *vt34* as a truncation of the RUNX transcription factor ortholog, RNT-1, a protein previously implicated in body size regulation and specification of cells of the male tail [23]. We demonstrate that *vt34* also has male tail defects and that these perturbations can be rescued by RNT-1 overexpression in *vt34* animals. Although *rnt-1* mutants have reductions in *dat-1* mRNA expression, rescue of *vt34* Swip by dopaminergic expression of *rnt-1* is incomplete, suggesting contributions from expression in other neurons or muscle cells. Indeed, we find that Swip of *vt34; dat-1* double mutants exhibit additivity, and *vt34* animals demonstrate altered sensitivity to both aldicarb and levamisole, consistent with both DA-dependent reductions in motorneuron acetylcholine release and postsynaptic changes of muscle acetylcholine receptor signaling capacity.

## Methods

### *C*. *elegans* strains and husbandry

*C*. *elegans* strains were cultured on OP50 or NA22 bacteria and maintained using standard methods and conditions [24]. Unless constructed in house or otherwise noted, strains were provided by the *Caenorhabditis* Genetics Center, which is funded by the NIH Office of Research Infrastructure Programs (P40-OD101440). The wild type strain is *N2* Bristol. The *dat-1(ok157)* strain was a gift of J. Duerr and J. Rand (Oklahoma Medical Research Foundation, Oklahoma City).

### Plasmids, transgenic animal production, and genetic crosses

P_*dat-1*_:GFP::DAT-1 and [25], P_*dat-1*_::GFP [26] [20], were described previously. The *rnt-1* expression construct (P_*rnt-1*_::GFP, pHK193) was a kind gift from Dr. Yugi Kohara [27]. The fosmids WRM0621aD07 and K06A5 were obtained from SourceBioscience. The plasmid for DA-specific rescue of *rnt-1* was generated using Gibson Assembly (New England Biolabs). A gateway vector containing P_*dat-1*_::mcherry with an *unc-10* 3’UTR (Addgene) was amplified and combined with *rnt-1* cDNA amplified from a N2 cDNA extract to generate P_*dat-1*_::*rnt-1*::mCherry::*unc-10 3’UTR*. The plasmid was verified by fluorescent Sanger sequencing (Vanderbilt Technologies for Advanced Genomics) prior to injection.

Animals carrying extrachromosomal arrays were created by co-injecting transgenes using methods described previously [28]. All transgenes were fully sequenced prior to injections. For transgenic rescue experiments, genomic regions plus at least 1kB upstream of the start of the transcript were amplified with a high-fidelity polymerase (KAPA hifi hotstart ready mix, KAPA Biosystems) and purified prior to injection. *rnt-1* rescue constructs were injected into *vt34; him-5(e1467)* animals. The fosmid WRM0621aD07 and the PCR product were both injected at 20 ng/μL, along with P_*unc-122*_::RFP and P_*dat-1*_::mCherry. The *gst-43* PCR product (10 ng/μL), *pamn-1* PCR product (40 ng/μL), and the *nmtn-1* fosmid K06A5 (40 ng/μL) were injected onto the *vt34* background with the markers P_*unc-122*_::RFP and P_*dat-1*_::mCherry for a total concentration of 100 ng/μL. pKH193 (P_*rnt-1*_::GFP) was injected into N2 animals at a concentration of 50 ng/μL) with P_*dat-1*_::mCherry (20 ng/μL) and pBSKII (40 ng/μL).

All genetic crosses were performed using integrated fluorescent reporter strains to mark chromosomes in trans. As matings of *vt34* or *rnt-1* males are not successful, crosses were performed with *vt34* or *rnt-1* hermaphrodites and males of the other genotype involved. Single worm PCR was conducted to analyze a deletion and crosses involving *vt34* mutations were analyzed by fluorescent Sanger sequencing (Vanderbilt Technologies for Advanced Genomics).

### SNP mapping and whole genome sequencing

Mapping of the mutant locus was performed as described previously [29, 30]. In brief, *vt31* and *vt34* were outcrossed 4 times to N2 and then crossed to the CB4586 (Hawaiian) strain. Bulk segregate analysis was performed to determine the linkage group (LG) by generating lysates from Swip-positive and Swip-negative F2s and performing 96 well PCR using published primer sets. Experiments were replicated at least twice before determination of the LG and finer mapping by cloning individual Swip-positive F2s. Forty to fifty F3 worms were Swip tested at least four times. Lysates from individual clones was then used to PCR specific intervals along with linkage group to determine the Bristol island likely to harbor the Swip-inducing mutation.

For whole genome sequencing, genomic DNA from *vt31*, *vt34* and the parental strain (BY200) was isolated and analyzed as described previously [30, 31]. Samples were submitted for Illumina sequencing (Vanderbilt University VANTAGE Core). Mutations present in *vt31* and *vt34* and absent in BY200 were identified and those located on the mapped interval were chosen for further analysis.

### Swip assays

Swip assays were performed as previously described [20]. Briefly, synchronized nematode populations were generated by hypochlorite treatment and L1 animals were plated onto OP50 seeded plates. Early- to mid-stage L4 animals were identified by characteristic morphology and ten to fifteen worms were placed into 100 μL of water in a Pyrex Spot Plate (Fisher) with the number of worms paralyzed determined visually after ten min. Data represents at least 24 trials. In the case of transgenic lines, fluorescent positive animals at L2 or L3 stage were picked onto plates and placed at 12°C to mature to the L4 stage. All assays were performed blinded. Automated analysis of Swip was performed as described previously [32]. Individual L4 animals were placed in 20 μL of water of a Pyrex Spot Plate (Fisher) and 10 min movies (uncompressed AVI format) were created and analyzed using SwimR’s default settings without outlier exclusion [32].

### Male response assay

Mating assays to assess male responses to the presence of hermaphrodites was performed as previously described [33]. Five males of a single genotype were placed on a lawn with twenty *unc-31(e169)* hermaphrodites and the number of males that successfully responded to the presence of hermaphrodites within four min was recorded. A response was deemed successful if the male made flush contact with the hermaphrodite and initiated scanning along its body.

### Body length measurement

Body length measurements were performed as previously described [23], with slight modification. Adults were allowed to lay on plates for 3–5 hrs at 20°C. Embryos were aged 90 hrs and then placed on a freshly prepared 2% agarose pad and immobilized using 225 mM 2,3 butanedione monoxime (Sigma-Aldrich) and 2.5 mM levamisole (Sigma-Aldrich) in 10 mM HEPES. The full length of the animal was captured and quantified using the Straighten plugin in Image J (NIH).

### Sensitivity to exogenous DA

DA sensitivity assays were performed as previously described [34] using L4 animals. Briefly, ten L4 animals were transferred to 1.7% agar plates containing various concentrations of DA in 2 mM glacial acetic acid. Plates were used the same day they were prepared. Animals were acclimated to the plates for 20 min, and then scored as paralyzed or moving. Moving was defined as any animal capable of generating a body bend through a minimum or maximum amplitude. Assays were performed blind to genotype.

### DA measurement by HPLC

DA levels were measured as previously described [30, 35]. Briefly, a synchronized population of L4 worms were washed, frozen and then sonicated in a buffer containing EDTA. Protein content was determined using a BCA assay (Pierce), after which 5 ng/μL isoprotenol was added to the sample and DA was extracted by alumina column (Bioanalytical Systems, followed by analysis by HPLC (Waters) with electrochemical detection with the amperometric detector (oxidation: 0.5). Data was acquired by Millennium 32 software.

### 6-OHDA neurodegeneration assay

Sensitivity of DA neurons to 6-OHDA was performed as described previously [19, 20, 26]. Briefly, lines were crossed to the P_*dat-1*_::GFP strain *vtIs*7 to mark DA neurons. Synchronized L1 worms were grown on NA22 plates seeded with 8P bacteria until the L2-L3 stage. Worms were washed from the plate and were incubated with 7 or 10 mM ascorbic acid with or without 25, 35, or 50 mM 6-OHDA, for 1 hr in the dark on a nutator. Treated worms were plated on NA22 plates for 3 days at 20°C until the worms became adults. Fifty worms were then examined using a dissecting microscope (Zeiss) and scored for degeneration based on a four-point scale (where 4 = all CEP dendrites intact and 0 = complete loss of CEP dendrites). For scoring, worms were placed on fresh 2% agarose pads and immobilized using 225 mM 2,3 butanedione monoxime (Sigma-Aldrich) and 2.5 mM levamisole (Sigma-Aldrich) in 10 mM HEPES. Experiments were performed in triplicate.

### Confocal imaging and quantification of DAT localization

*In vivo* imaging of DAT-1::GFP was performed as previously described [20]. Briefly, transgenic animals were placed on a freshly prepared 2% agarose pads and immobilized using 225 mM 2,3 butanedione monoxime (Sigma-Aldrich) and 2.5 mM levamisole (Sigma-Aldrich) in 10 mM HEPES. A Z-stack of images was obtained using a Zeiss LSM 510 or 710 in the Vanderbilt Cell Imaging Core. By setting the gain of confocal illumination of synaptic regions just below the maximal gain of the microscope, we obtain the relative intensity of signal from cell soma and dendritic regions within the same animal. For quantification, imaging stacks obtained containing cell body, dendrites and synaptic regions were thresholded, and pseudo-colored using Metamorph (Molecular Devices, Sunnyvale, CA). Pixel densities were calculated after selecting the area of interest and then obtaining the average fluorescence intensity, with these values used to calculate the ratio of fluorescence in the synapse or dendrite relative to the cell body in the same animal. A minimum of ten animals per genotype was used for analysis.

### RNA isolation and qRT-PCR

Total RNA was extracted from L4 worms grown on NA22 plates. Briefly, worms were washed several times with M9 and rocked for two hrs to clear bacteria. Worms pellets were frozen at -80°C and then ground with a mortar and pestle in liquid nitrogen. Trizol (Life Technologies) was added to the worm powder, and centrifuged for 10 min at 14,000 rpm 4°C. Chloroform was added to the supernatant, which was centrifuged to localize the RNA to the upper aqueous phase. The upper phase was removed and precipitated using isopropanol and ethanol. qRT-PCR was performed using the KAPA SYBR FAST One-Step qRT-PCR Kit (KAPA Biosystems) and analyzed on the Eco Real-Time PCR system (Illumina). Primers were validated using total RNA in varying amounts to generate a standard curve. Only primer sets that had an R^2^ of at least 0.9 were used. Sample purity was verified by omitting the RT enzyme from the master mix. The following primer sets were used: *pmp-3* (forward: 5’-GTTCCCGTGTTCATCACTCAT-3’, reverse: 5’-ACACCGTCGAGAAGCTGTAGA-3’), *cat-1* (forward: 5’-AAGCGTGTGAAACGTCAGTG-3’, reverse: 5’-CGGGTTCTCGTCCCAATCAA-3’), *cat-2* (5’-forward: TTGCGGTATATGCGGAGGC-3’, reverse: 5’-ACTTTGGTGGCGATGACTGT-3’), *dat-1* (forward: 5’-GACTCTACCCGGATGGCAAA-3’, reverse: 5’-AGACCGATGGTCTCTTGAGC-3’). Each reaction was carried out in triplicate, and the Ct values were averaged. As described previously [36], any samples with standard deviations that were >0.5 Ct units were re-run until the standard deviation between replicate values was less than 0.5. Ct. Values were converted to a linear range by calculating 2^-Ct^. Test genes were normalized to the control gene, *pmp-3*.

### Aldicarb and Levamisole assays

Aldicarb sensitivity assays were carried out following the protocol of Mahoney and colleagues [37]. Aldicarb (Chem Service Inc., West Chester, PA) was prepared as a stock solution dissolved at room temperature in 70% ethanol to a concentration of 100 mM, and was used to make NGM agar plates with a final concentration of 1 mM aldicarb. Genotypes were blinded and 25–30 young adult animals were transferred to aldicarb plates. We scored animals for total number moving every 30 min for 5 hrs. Worms were considered paralyzed if they no longer respond to tapping on the head for three times. The assay was performed blindly three times over three different days. Levamisole sensitivity assays were carried out similarly to the aldicarb assays and following the protocol of Gottschalk and colleagues [38]. OP50 plates were prepared with 1.5mM levamisole and 20–30 animals were scored per day for 4 days. Worms were counted every 5 min until they stopped responding. The assay was performed blindly four times over four different days.

## Results

### Dopaminergic characteristics of *vt31* and *vt34*

In our forward genetic screen to identify novel determinants of DA signaling [30], we identified two mutant lines, *vt31* and *vt34*, that display a reserpine-sensitive Swip phenotype similar to *dat-1* null animals, though they do not harbor mutations in *dat-1*. In preliminary studies, we found that *vt31* and *vt34* fail to complement, suggesting that their mutations lie within the same gene. Indeed, in efforts described later in this report, the mutation responsible for Swip in both lines mapped to the same genomic region and was later found to arise from the same substitution. Due the non-clonal nature of our screen, we conclude that both mutations arose from the same founder animal. Therefore, subsequently in our report, we only present our studies with the *vt34* line.

**Fig 1:**
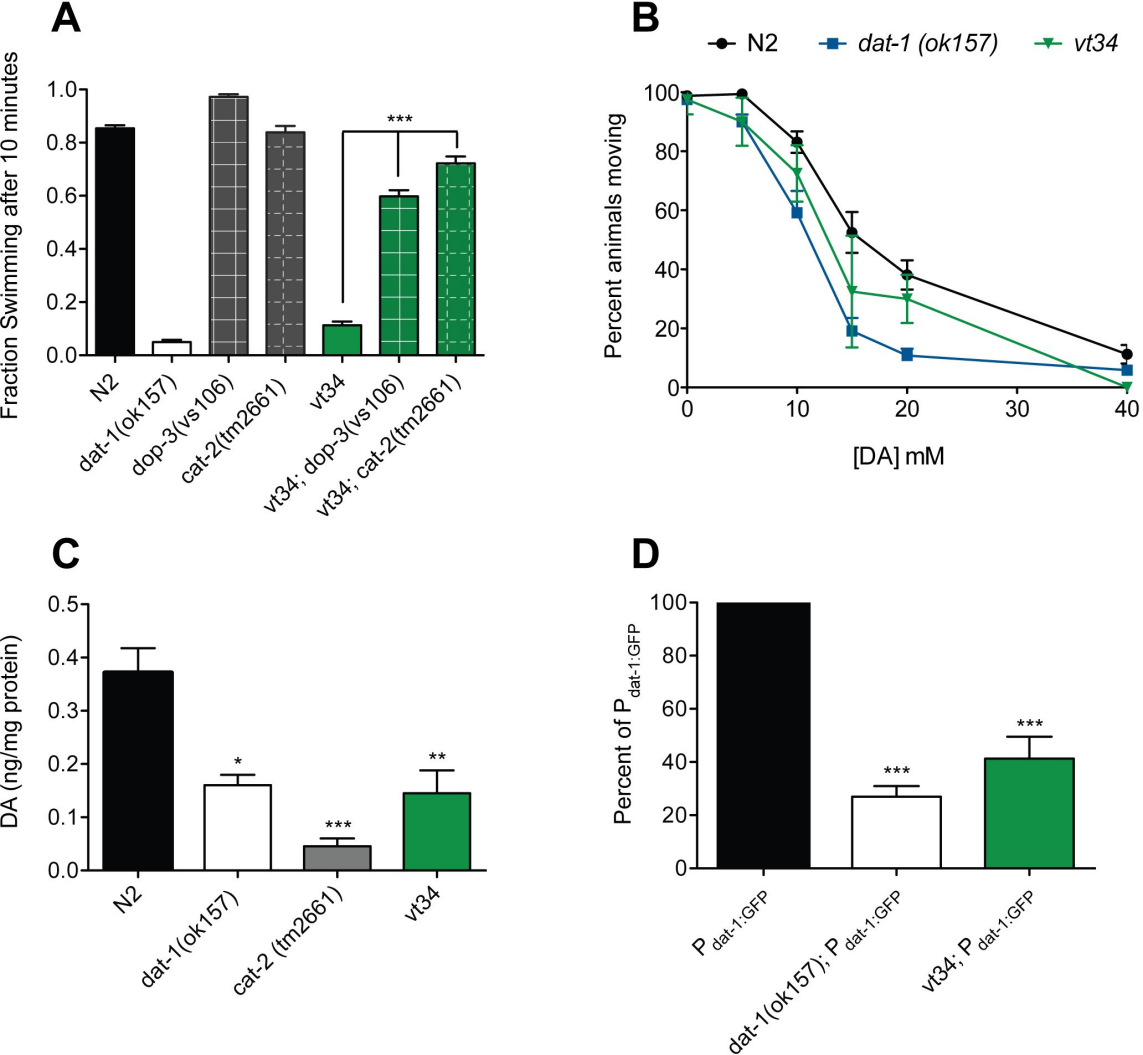
*vt34* exhibits DA-dependent behaviors that parallel *dat-1* mutants. (A) *vt34* Swip is dependent on DOP-3 and CAT-2. At least 10 L4 animals per well were assayed in water and scored as animals swimming over total number of animals after 10 min. Data represents at least 24 trials. Data were analyzed using one-way ANOVA with multiple Bonferroni post-tests with * = *P* < .05, * = *P* < .01, and *** = *P*<0.001. Error bars represent SEM. (B) *vt34* displays slightly enhanced sensitivity to exogenous DA on solid substrate. Sensitivity to exogenous DA is equivalent in N2 and *vt34* animals. Movement on plates was assessed as described in Methods. Data were analyzed using a two-way ANOVA and revealed a significant genotype x drug interaction (*P* < .001). Comparison of movement rates at individual concentrations, assessed via Student’s t-tests, revealed a significant difference between *dat-1* and N2 (*P* < 0.001) at 10, 15, and 20 mM DA; *vt34* did not demonstrate significant differences with N2 at any concentration. Data represents at least 8 tests with 10 animals per strain per concentration. (C) *vt34* mutation results in a reduction of DA levels. DA levels were measured by HPLC in synchronized L4 animals as described in Methods. DA levels in *dat-1* and *vt34* animals are intermediary between N2 and *cat-2* animals. Data represents at least three biological replicates. Data were analyzed using one-way ANOVA with multiple Bonferroni post-tests with * = *P* < .05, * = *P* < .01, and *** = *P*<0.001. Error bars represent SEM. (D) *vt34* exhibits decreased sensitivity to DA neuron degeneration induced by 6-OHDA treatment. Treatment with 6-OHDA and degeneration scoring were performed as described in Methods. Data are represented as percent of N2 and represents at least 50 animals scored in three different experiments. Data were analyzed using one-way ANOVA. *** = *P*<0.001 and error bars represent SEM.

Reserpine-sensitive Swip is dependent upon excess DA action at the DA receptor DOP-3 [20]. To corroborate the dependence of *vt34* Swip on DA signaling, we generated a *vt34* double mutant using *cat-2-(tm2661)*, a loss of function mutation in the *C*. *elegans* ortholog of the DA biosynthetic enzyme tyrosine hydroxylase, as well as using *dop-3(vs106)*, a loss of function mutant in the D2-type DA receptor that mediates Swip. As shown in Fig 1A, both double mutant lines demonstrated significantly greater swimming activity than *vt34* animals, consistent with *vt34* Swip as dependent on DA production and signaling.

Previously, we demonstrated that *dat-1* mutants display increased slowing in the presence of exogenous DA, compared to N2, in assays of movement on solid surfaces [30], likely a result of diminished DA clearance that could otherwise limit locomotor inhibition. Consistent with these findings, when we exposed *dat-1(ok157) mutants* to plates containing different concentrations of DA, we detected a leftward shift of the dose-response curve and a decreased DA EC_50_ comparing *dat-1* to N2 (*dat-1* EC_50_ = 8.5 +/- 0.9mM, N2 EC_50_ = 17.9 +/- 1.3mM) (Fig 1B). Studies of locomotor sensitivity to DA using *vt34* animals also suggested a leftward shift relative to N2, intermediate to effects seen with *dat-1*, though EC_50_ difference with N2 did not emerge in post-tests (*vt34* EC_50_ = 11.5+/- 1.1mM). These data indicate that the Swip phenotype of vt34 animals does not derive from a reduction in DAT-1 levels or activity, though caution is needed since the higher activity in swimming assays likely drives significantly greater DA release and increased demand for DA clearance.

*Dat-1* mutant animals demonstrate a significant reduction in DA levels compared to N2 [18], findings we reproduced in this study (Fig 1C). Reduced brain DA levels are observed in DAT KO mice [39], believed to arise from a failure to recycle and store the neurotransmitter. We also found DA levels to be decreased in *vt34* relative to N2 with a comparable reduction to that observed with *dat-1* animals. As a control, we demonstrated that DA levels are virtually eliminated in animals that cannot synthesize DA via the *C*. *elegans* ortholog of tyrosine hydroxylase (CAT-2), the rate limiting step in neuronal DA synthesis. Together, these findings are consistent with reductions of DA synthesis or vesicular storage capacity and/or a reduction in DAT-1 mediated DA recycling.

Finally, to assess DAT-1 functional capacity *in vivo*, we examined the ability of 6-OHDA to induce DA neuron degeneration in *vt34* animals. Previously [19], we demonstrated that 6-OHDA induced degeneration of DA neurons is dependent on the expression of DAT-1, consistent with the transporter’s role in driving intracellular accumulation of the neurotoxin. As shown in Fig 1D, *vt34* animals expressing an integrated DA neuron-specific transcriptional reporter (P_*dat-1*_:GFP), supported 6-OHDA induced degeneration of DA neurons comparable to that seen in DAT-1 deficient animals. Together, these experiments provide evidence that a significant component of the Swip of *vt34* animals likely derives from changes in expression or activity of DAT-1.

### Evidence that *vt34* Swip involves more than reduced DAT-1 capacity

**Fig 2:**
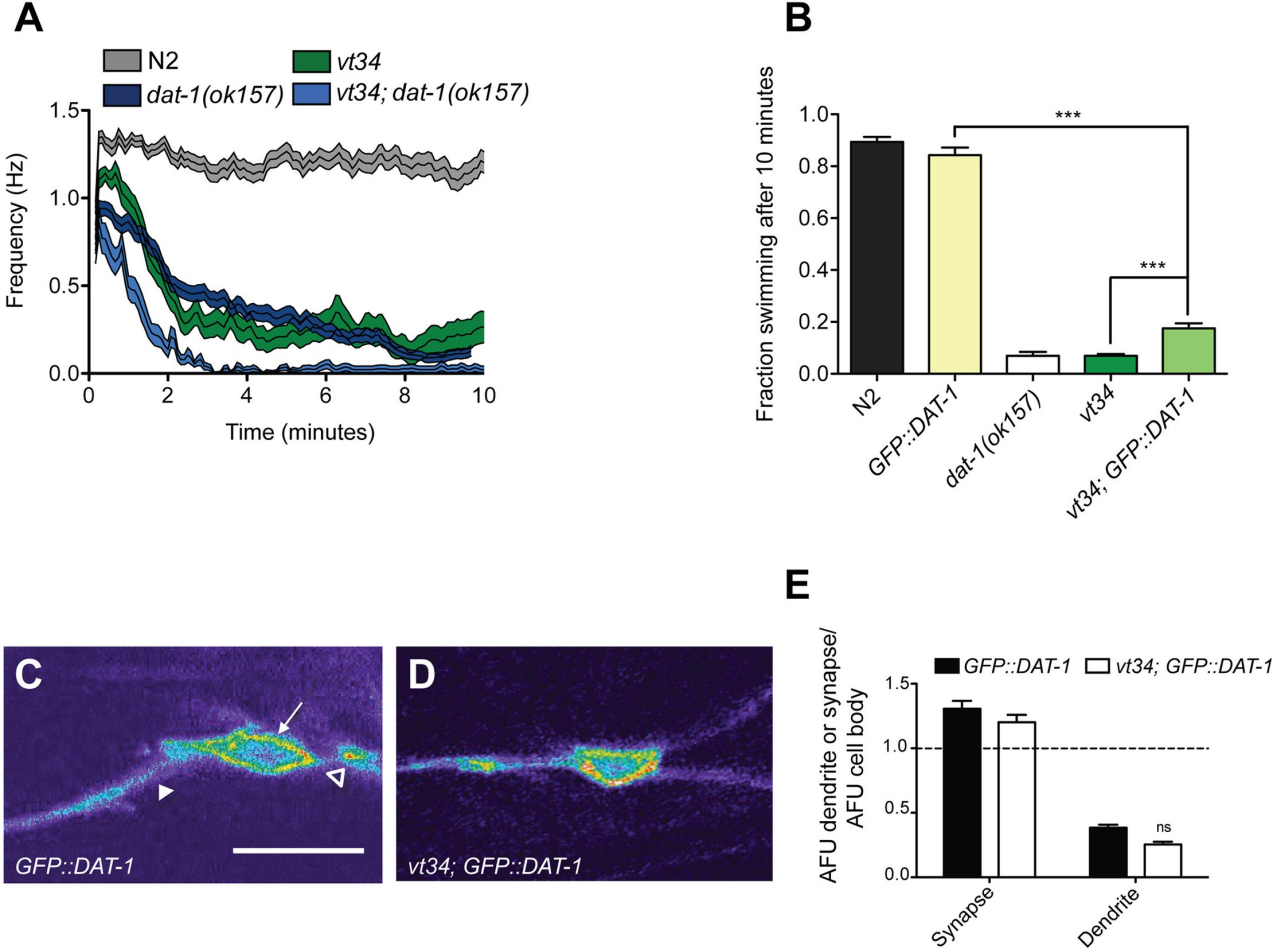
*vt34* acts in a parallel pathway to reduce DAT-1 activity. (A) Automated analysis of Swip reveals enhanced Swip relative to *dat-1* in *vt34; dat-1* double mutants. Single L4 animals were recorded using a video capture system and analyzed with SwimR software as described in Methods. Swimming frequency with SEM shaded above and below the mean is displayed. Data represent at least 20 animals per genotype analyzed by two-way ANOVA with multiple Bonferroni post-tests. Significant genotype x time interaction effects were evident with post-tests indicating *P* < 0.001 for N2 vs. *vt34*, *dat-1(ok157)* vs. *vt34; dat-1(ok157)*, and *dat-1(ok157)* vs. *vt34; dat-1(ok157)*. (B) Over-expression of GFP::DAT-1 does not fully rescue *vt34* Swip. Transgenic animal production and manual endpoint assays of swimming behavior using L4 animals were pursued as described in Methods. In these assays, 10 animals were assayed per well and at least 24 assays were performed per genotype. Data were analyzed using one-way ANOVA with multiple Bonferonni post-tests. *** = *P* < 0.001 and error bars represent SEM. (C, D) *vt34* animals expressing P_*dat-1*_:GFP::DAT-1 exhibit normal DAT protein distribution as visualized *in vivo* using confocal microscopy. Single planes containing cell body (white arrow), synapse (open triangle) and dendrites (closed triangle) were imaged and quantified as described in Methods and Materials. Anterior is left, and represents ratiometric image of a CEP neuron. Scale bar represents 20 μm. (E) Quantification of GFP::DAT-1 fluorescence levels from images as in C and D, expressed as a ratio of synapse/cell body or dendrite/cell body in individual animals as described in Methods, analyzed by two-way ANOVA followed by Bonferroni post-tests. No significant genotype x region effect was observed nor were the individual compartments statistically different. Data derive from at at least 12 animals per genotype. Error bars represent SEM.

To this point, we had analyzed the Swip phenotype at a single time point, scoring results by eye. If the mutation of *vt34* induces Swip by altering DAT expression or function, an epistasis experiment combining *vt34* and *dat-1* mutations should confirm this possibility. The strong Swip phenotype of both *dat-1* and *vt34* at 10 min, however, makes epistasis experiments difficult when using endpoint observations. To gain better insight into the genotype differences of single and double vt34 mutants, we implemented an automated approach [32, 40] that allows for an assessment of the time-dependence of worm movement. As expected from endpoint assays, across the recorded time of 10 minutes, N2 animals maintained a thrashing frequency between 1 and 1.5 Hz. In contrast, both *dat-1(ok157)* and *vt34* animals quickly slow (comparably) to a frequency below 0.5 Hz within the first two min of analysis (Fig 2A). In contrast, *vt34; dat-1(ok157)* double mutants demonstrate a more rapid paralysis then either single mutant. This additive pattern suggests that while *vt34* may impact DAT-1 expression or function, it also appears to drive paralysis via a parallel pathway. This suggests that the comparable paralysis seen comparing *dat-1* and *vt34* mutants may be due to loss of DAT-1 in the latter mutants to a level somewhat less than observed in a full deletion, combined with the effects of another negative modifier of swimming behavior.

If the Swip of *vt34* derives from a reduction in DAT-1 expression or function, we reasoned that transgenic expression of DAT-1 in DA neurons should rescue paralysis. Support for this idea derives from our prior finding that over-expression of a GFP::DAT-1 fusion protein can rescue Swip present in *dat-1* and *swip-13(vt32)* mutants [20, 41]. Using *vt34* animals in which a GFP::DAT-1 translational fusion protein, driven by the *dat-1* promoter, was expressed (Fig 2B), we indeed observed a significant rescue of Swip activity. However, the magnitude of this rescue was considerably more modest that that previously observed for Swip exhibited by *dat-1* and *swip-13* mutants, despite visual confirmation of transgene expression (Fig 2C), use of multiple DNA preparations, and the testing of multiple DNA concentrations for transgene expression, suggesting that, as noted above, DAT-1 independent mechanisms contribute to *vt34* Swip.

One possibility for the incomplete ability of GFP::DAT1 to rescue the Swip of *vt34* animals is an impact of the *vt34* mutation on transporter trafficking, limiting export of the fusion protein to synapses. Indeed, our prior imaging studies [20, 42] demonstrated perturbed synaptic trafficking of C-terminal truncations of GFP:DAT that also fail to rescue the Swip and 6-OHDA insensitivity of *dat-1* mutants. As previously described, quantitation of the distribution of GFP:DAT-1 in N2 head DA neurons demonstrates that expression of GFP::DAT-1 is highest in synapses, followed by the cell soma and then in dendrites (Fig 2C and 2D). In comparing these patterns with GFP::DAT-1 fusion protein expression in *vt34* animals, we detected no change in transporter distribution. We conclude that the ability of *vt34* to induce Swip and reduce 6-OHDA sensitivity is unlikely to be mediated by grossly perturbed trafficking of DAT-1 to synapses.

### *vt34* has a truncation mutation in the RUNX transcription factor ortholog RNT-1

**Fig 3:**
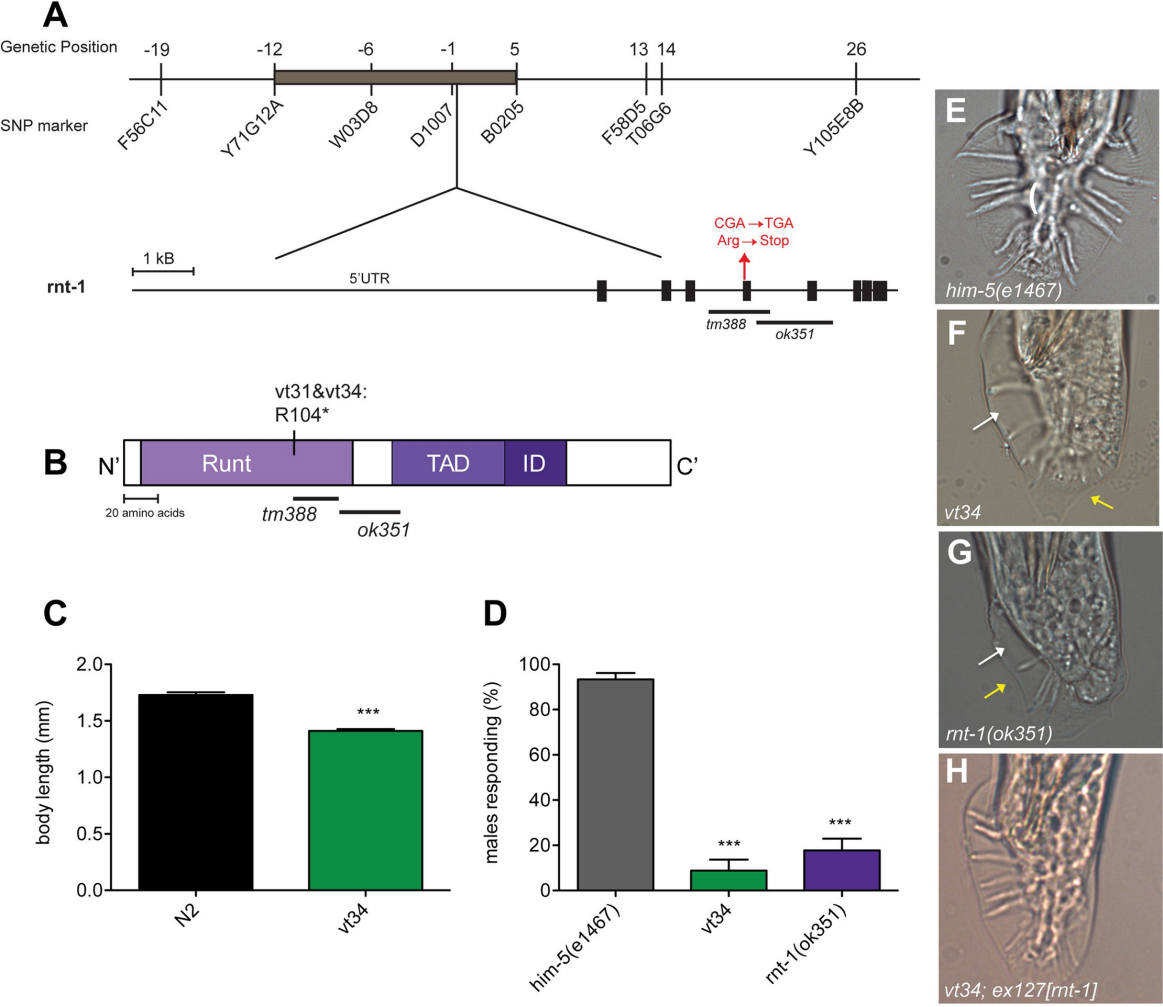
*vt34* harbors a mutation in the RUNX homolog *rnt-1*. (A) SNP mapping narrowed down the mutation responsible for Swip to the middle of LG1. Whole-genome sequencing identified a C → T mutation in *rnt-1* that results in mutation from arginine to a stop codon; deletion alleles *tm388* and *ok351* are also located in that same region. (B) Schematic of the RNT-1 protein. RNT-1 contains a highly conserved DNA binding domain (Runt), a transactivation domain (TAD) and an inhibitory domain (ID). *vt31* and *vt34* contain a STOP codon near the end of the RUNT domain (C) *vt34* animals, like *rnt-1* animals, have reduced body size. Synchronized L1’s were aged for 90 hrs before measuring the body size. Data represent a mean of least ten animals; error bars represent SEM. *** = *P* < .01, two-tailed Student’s t-test (D) *vt34* males fail to respond to hermaphrodites in the first step of mating. Animals of a genotype in a *him-5(e1467)* background were placed on a bacterial lawn with *unc* hermaphrodites. The number of males that initiated scanning in four min was recorded. Data represent the mean of behavior of five males in at least three independent experiments and was analyzed using one-way ANOVA with multiple Bonferroni post-tests. *** = *P* < 0.001. Error bars represent SEM. (E-H) Representative images of male tails from N2 and mutants. N2 animals (E) have a rounded fan with 9 characteristic rays on either side. *Rnt-1* and *vt34* mutant males (F, G) display irregularly shaped fans (yellow arrows) and missing or fused rays (white arrows). Transgenic expression of genomic *rnt-1* is able to rescue *vt34* male tail morphology (H).

To identify the gene harboring the Swip-inducing mutation in *vt34*, we first used SNP mapping techniques to identify the rough genomic location of the *vt34* mutation [29]. We found that the Swip of *vt34* (and *vt31*) animals maps to the middle left region of LG1 (Fig 3A). We then performed whole genome sequencing on *vt31*, *vt34*, and the parental strain [43]. We filtered the datasets for SNPs in the mapped region to identify mutations found in both *vt31* and *vt34* but not found in the parental strain. In this analysis, we identified 15 genes bearing mutations, with 12 verified by Sanger Sequencing (S1 Table). We found the mutation in *rnt-1*, the single ortholog of the RUNX transcription factor family [23], likely to be a functional variant as the mutation represents a nonsense substitution at the start of the gene’s fourth exon (Fig 3A and 3B) that encodes the highly conserved Runt DNA binding domain. Studies of mammalian RUNX proteins [44, 45] show that a full Runt domain is required for DNA binding, as well as for the binding of the RUNX partner CBFβ (BRO-1 in *C*. *elegans*) [46, 47].

### *vt34* exhibits phenotypes characteristic of *rnt-1* mutants

To seek confirmation that the *rnt-1* mutation identified in our sequencing efforts is functional, we first asked whether phenotypes characteristic of *rnt-1* loss of function mutants are observed *in vt34*. The namesake phenotype for *rnt-1* is a decrease in body size [23]. We measured the body length of N2 and *vt34* animals from a synchronized population that were aged to young adults. We found *vt34* mutant to be significantly (~20%) smaller than N2 animals, consistent with previously identified *rnt-1* mutants (Fig 3C) [23]. *Rnt-1* mutants also exhibit defects in the structure of the male tail, as *rnt-1* is required for proper proliferation of sensory ray precursor cells [27]. The wildtype male tail consists of a round fan with nine pairs of chemosensory rays and a pair of spicules- the copulatory structure. These rays, three of which are dopaminergic, are responsible for sensing the hermaphrodite and the initiation of scanning for the vulva [48, 49]. Consistent with this phenotype, homozygote *vt34* males were unable to produce successful matings, and so we performed a male response assay to assess the ability of males to move toward and engage hermaphrodites [33]. We generated a larger population of *vt34* males using the *him-5(e1467)* background and found that whereas almost 100% of N2 males responded to hermaphrodites, initiating the process of vulval scanning within 4 min, males with a deletion in *rnt-1* (*rnt-1(ok351*)) downstream of the *vt34* mutation failed to respond, nor did *vt34* males (Fig 3D). Next, we examined the morphology of *vt34* male tails. In contrast to a circular fan shape of an N2 male tail (Fig 3E), we found that both *vt34* and *rnt-1(ok351)* tails to be irregularly shaped, with several rays missing (Fig 3F and 3G). The specific rays that were missing varied from animal to animal, and there appeared to be no correlation with their identity and the three pairs of dopaminergic rays. Importantly, transgenic *vt34* animals expressing the *rnt-1* genomic sequence demonstrated normal tail morphology, verifying that the tail defect in *vt34* arises from mutation of *rnt-1 (*Fig 3H*)*.

### *rnt-1* mutants exhibit DA-dependent Swip

**Fig 4:**
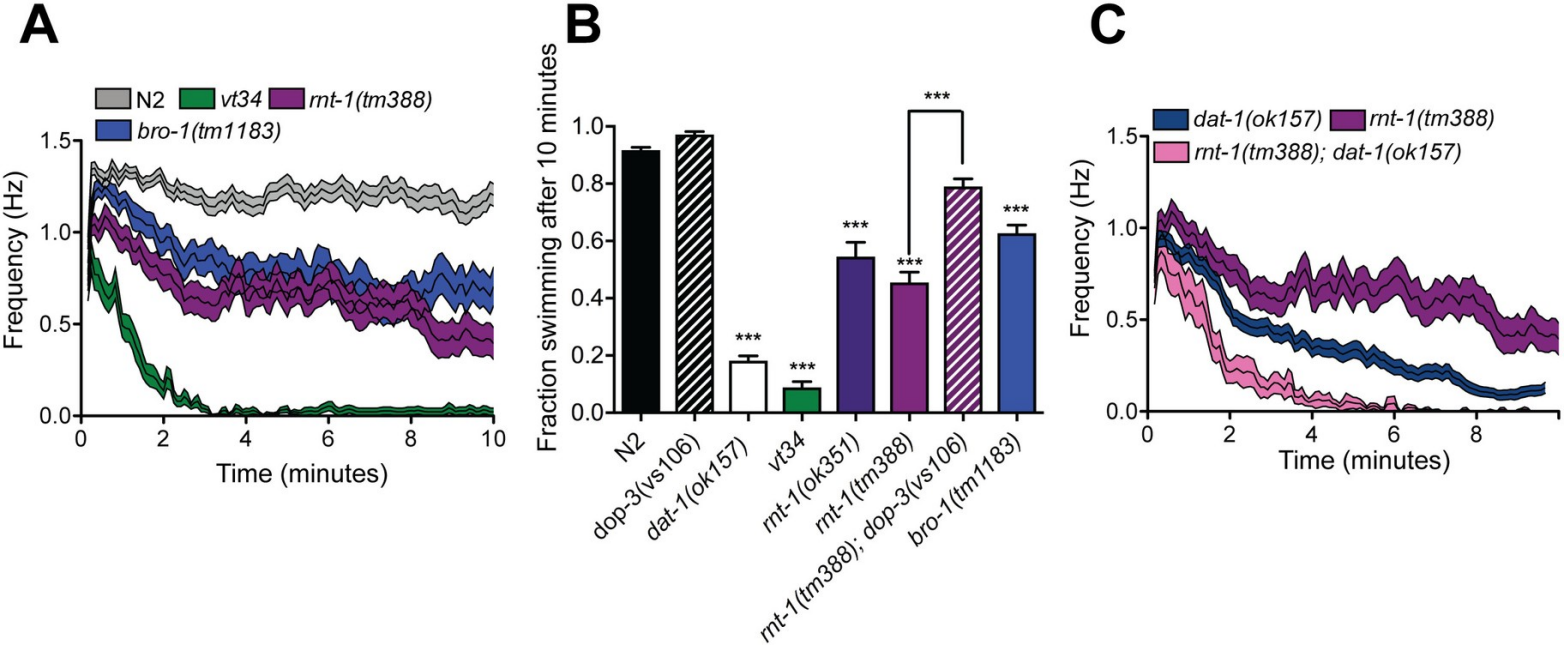
*rnt-1* mutant exhibits both DA-dependent and independent Swip. (A) *rnt-1* and *bro-1* mutants exhibit intermediate Swip as compared to N2 and *vt34*. Data were graphed as mean frequency with SEM shaded above and below. Data represent at least 20 animals per genotype analyzed by two-way ANOVA with multiple Bonferroni post-tests. *P* < 0.001 for all genotype combinations. (B) The Swip in *rnt-1* mutants is DOP-3 dependent. At least 10 L4 animals per well were assayed per trial as described in Methods. Data represents at least 24 trials. Data were analyzed using one-way ANOVA with multiple Bonferroni post-tests. *** = *P* < 0.001 compared to N2 animals and error bars represent SEM. (C) Automated analysis of Swip reveals enhanced Swip in *rnt-1; dat-1* double mutants. Mean frequency of swimming rates was graphed with SEM shaded above and below. Data represent at least 20 animals per genotype and were analyzed by two-way ANOVA with multiple Bonferroni post-tests. *P* < 0.001 for all genotype combinations.

Next, we sought to examine the relationship between *rnt-1* and DA signaling, first examining the swimming behavior of *rnt-1(tm388)*, a deletion whose location is the closest to the *vt34* point mutation (Fig 3A and 3B). We also examined the swimming behavior of animals with a loss of function mutation in *bro-1*, a known binding partner of RNT-1. BRO-1 does not bind to DNA itself but binds to RNT-1, enhancing its partner’s DNA binding activity [46]. Using both automated and endpoint analyses, we observed that *rnt-1(tm388)* animals exhibit Swip, swimming at a frequency intermediate between wildtype and *vt34 (*Fig 4A and 4B*)*. Interestingly, the *bro-1(tm1183)* deletion mutant also demonstrated significant Swip (Fig 4A and 4B), quantitatively comparable to that of *rnt-1(tm388)*. In manual endpoint assays, we found that another *rnt-1* allele, the deletion mutant *rnt-1(ok351)*, paralyzed at levels like *rnt-1(tm388)*, being more active than seen with *vt34* mutants (Fig 4B). This suggests the possibility of dominant-negative or gain of function actions of the *vt34* mutation that are lacking in the *rnt-1* deletion mutants, actions of *vt34* that would not be available to *rnt-1* deletion mutants. Importantly, the Swip of *rnt-1(tm388)* was significantly rescued by mutation of *dop-3* (Fig 4B). Lastly, we examined the relationship between *rnt-1* and *dat-1* with respect to Swip. As we demonstrated with the *vt34; dat-1* double mutants, *rnt-1; dat-1* double mutants demonstrate Swip significantly more than either mutation alone (Fig 4C). Together these data indicate that the DA-dependent Swip of *vt34* is the result of a defect in the DNA binding activity of RNT-1, and that RNT-1 drives Swip via excess DA signaling as well as a parallel pathway.

### RNT-1 is expressed in DA neurons but is insufficient to rescue *vt34* Swip

**Fig 5:**
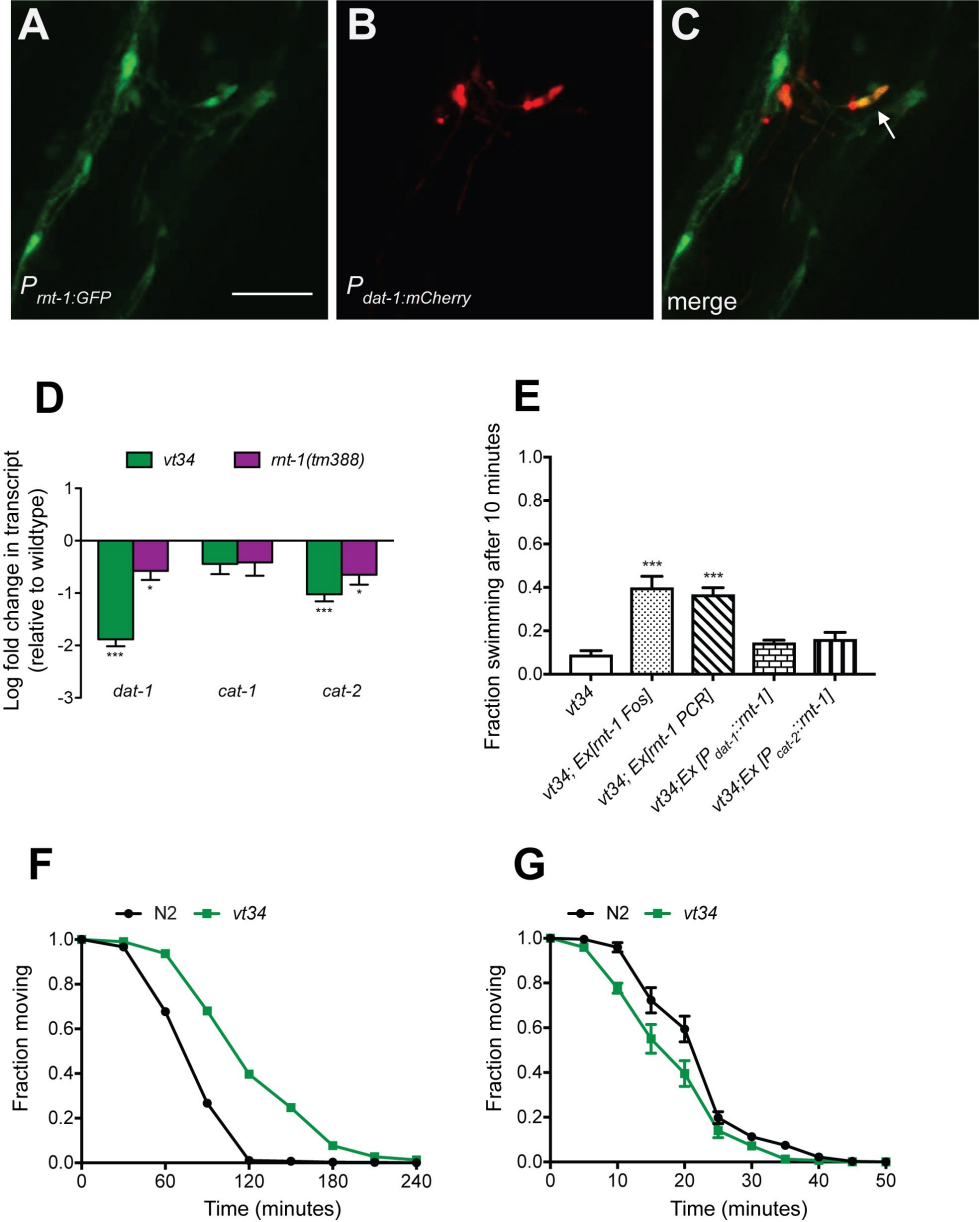
RNT-1 acts non-cell autonomously to influence expression of *dat-1* and *cat-2*. (A-C) GFP driven by the *rnt-1* promotor shows expression in DA neurons marked by P_*dat-1*_::mCherry. Representative confocal images of N2 transgenic animals. (D) Quantitative RT-PCR. Transcript levels of *dat-1* and *cat-2* are significantly decreased in *vt34* and *rnt-1(tm388)* animals. Data are shown as a log fold change relative to N2 animals and represents at least three technical and biological replicates. Data were analyzed by a Student’s t-test. *** = *P* < 0.001. Error bars represent SEM. (E) *rnt-1* expression by fosmid or PCR product significantly rescues *vt34* Swip. However, *rnt-1* driven by the *dat-1* promotor only modestly rescues *vt34* Swip and not at all, though we encountered more variability, with the *cat-2* promoter. Transgenic animals were assayed for Swip using manual endpoint assays. Progeny from three transgenic lines per experiment were tested. Data was analyzed by One Way ANOVA. *** = *P* < 0.001 and error bars represent SEM. (F) *vt34* exhibits reduced sensitivity to the acetylcholinesterase inhibitor aldicarb. Lines represents mean at each time point with errors bars representing SEM. At least 70 animals were tested per genotype. Data was analyzed by two-way ANOVA with Bonferroni post-tests with *P* values at 60, 90, 120, 150 and 180 <0.0001. (G). *vt34* exhibits increased sensitivity to the acetylcholine receptor agonist levamisole. Lines represent mean at each time point with error bars representing SEM. At least 80 animals were test per genotype. Data was analyzed by two-way ANOVA with Bonferroni post-tests with *P* values at 10, 15, 20 <0.0001.

RNT-1 is mainly expressed in the hypodermal seam cells and the male tail [50]. Tiling array data [51], however, suggests that *rnt-1* is expressed in L4 DAergic neurons, the stage at which we assay Swip [51]. In other studies, *rnt-1* has been reported to be expressed in unidentified head neurons [52]. To validate whether these findings represent the expression of *rnt-1* in DA neurons, we obtained a P_*rnt-1*_::GFP [27] transcriptional reporter construct and expressed it in N2 animals along with a marker for DAergic neurons (P_*dat-1*_::mCherry). Notably, we observed co-expression of these reporters (Fig 5A–5C).

To determine whether RNT-1 modulates DA signaling through its DNA binding activity, we quantified mRNA levels in L4 animals of the DA neuron-specific genes *dat-1*, and *cat-2*, as well as the monoamine vesicular transporter *cat-1*, expressed by both DA and 5-HT neurons. (Fig 5D). Compared to N2, *vt34* animals demonstrated a hundred-fold reduction in *dat-1* mRNA levels. The *rnt-1(tm388)* mutant also displayed a significant decrease in *dat-1* levels, albeit less severe than *vt34*. We found that *cat-1* levels were not significantly altered, but *cat-2* levels were significantly decreased in *vt34* and *rnt-1(tm388)* animals. These findings indicate that RNT-1 participates positively in the expression of multiple determinants of the dopaminergic phenotype including DA synthesis and re-uptake. Notably, the greater reduction of *dat-1* mRNA in the *vt34* animals compared to *rnt-1* indicates that the *vt34* mutant exhibits a dominant-negative action with respect to this measure that is not generated by *rnt-1* deletion.

Since we were able to rescue the male tail defect in *vt34* with *rnt-1* under its own promotor, we sought to rescue the Swip phenotype as well. As shown in Fig 5E, we obtained significant rescue of *vt34* Swip injected with a fosmid containing *rnt-1* or with *vt34* transgenic animals expressing a PCR product that contains the *rnt-1* promotor and downstream genomic regions. Dominant negative actions of *vt34* on the wildtype gene may explain the incomplete rescue obtained with these constructs (~40% of WT levels) or that the constructs achieve insufficient expression levels. Next, given that *rnt-1* is expressed in DA neurons and regulates DA gene expression, we generated *vt34* transgenic animals that expressed *rnt-1* cDNA driven by either the *dat-1* or *cat-2* promoter. We did not observe significant rescue with the *dat-1* or *cat-2* promoters. These findings indicate that cell-nonautonomous effects contribute significantly to the *vt34 rnt-1* mutation, possibly even more than cell autonomous effects. Tiling array mRNA expression studies indicate a significant expression of *rnt-1* in L2 body wall muscles (wormviz.inf.eth.ch). Possibly loss of *rnt-1* could act in muscle to reduce contraction ability, providing a parallel, synergistic pathway for Swip to that triggered by excess DA signaling acting on motorneurons to reduce ACh release. Therefore, we tested the sensitivity of *vt34* to aldicarb, a cholinesterase inhibitor that augments synaptic ACh levels and leads to desensitization of ACh receptors and muscle paralysis (Fig 5F). N2 and *vt34* young adults were treated with 1 mM aldicarb and the number of animals moving on plates was recorded. We found that *vt34* animals exhibited significantly reduced sensitivity to aldicarb (Fig 5G), consistent with either actions of the mutant to limit ACh release or to decrease ACh receptor activation to induce muscle contractions. To examine these options, we tested the mutant animals for their sensitivity to the direct nicotinic receptor agonist levamisole. We observed that vt34 animals are more sensitive than WT animals to levamisole, with a shift to the left of the levamisole dose-response curve for paralysis induction.

## Discussion

In this study, we have identified a novel role for the RUNX transcription factor ortholog, RNT-1, in regulating DA signaling. DA release and signaling requires the appropriate expression of biosynthetic enzymes (including *cat-2*), vesicular monoamine transporter (VMAT/*cat-1*), DA receptors, DA transporter, and a plethora of identified regulatory proteins. All of these participants are significantly regulated at the transcriptional level. We now identify RNT-1 as one of those transcription factors that contribute to DA signaling in *C*. *elegans*. As our SNP-mapping was based on the Swip phenotype alone, we tested other mutations within the mapped region (S1 Fig) and found no evidence that these mutations replicated the Swip of *vt34*. One other gene harbors a nonsense mutation, a neurofilament heavy peptide, *Y39G10Ar*.*15*. No alleles were available to test for Swip, so we generated transgenic *vt34* animals expressing a fosmid spanning the gene and did not see any significant rescue with this construct. Several genes are enriched in DA neurons, including *gst-43*, *pamn-1*, *nmtn-1*, and *maco-1*. We obtained available deletion lines and tested them for Swip, but none of them had a phenotype as severe as the *rnt-1* mutants we tested (S1 Fig), though we did see modest rescue with *pamn-1* and thus cannot fully rule out a synthetic phenotype contributed by this *rnt-1* linked gene.

RNT-1 and the rest of the RUNX family play important roles in development, including hematopoiesis, chrondrogenesis, and neurogenesis [53, 54]. Though RNT-1 is crucial to developmental events in *C*. *elegans* including the proliferation of seam cells, the architecture of the eight hermaphroditic DA neurons in *vt34* and other *rnt-1* mutant animals is visibly indistinguishable to N2, indicating that RNT-1 is not involved in the specification or differentiation of DA neurons.

A possible intersection between RNT-1 and DA signaling in *C*. *elegans* is the regulation of body size. RNT-1 influences body size through the TGF-beta signaling pathway, by interacting with SMA-4, which inhibits LON-1, a negative regulator of body size [23, 55]. The TGF-beta receptor DBL-1 seems to have two parallel pathways that impinge on LON-1, the other being action through SMA-6/DAF-4 to SMADs [55]. Interestingly, *cat-2* mutants are longer than wildtype animals of the same age, as are *dop-2* and *dop-3* mutants [56]. However, this effect is mediated by octopamine via receptors SER-3 and SER-6. It is thus unclear at this point whether there is crosstalk between the RNT-1/TGF-beta and DA pathways, though analysis of double mutants of *cat-2* with *sma-2*, *3*, *or 6* indicate a possible genetic interaction, though the interaction may arise cell nonautonomously [56]. However, more studies are needed to solidify this relationship.

We observed Swip in both *rnt-1* and *bro-1* mutants. As BRO-1 acts to enhance RNT-1’s DNA binding affinity, these findings suggest that Swip results from a loss of RNT-1’s transcriptional regulatory activities. Indeed, the highly conserved Runt domain of *rnt-1* is required for DNA binding. *In vitro* studies with human RUNX1 have demonstrated that the C-terminal end of the Runt domain is required for DNA recognition [57, 58]. CBFβ/BRO-1 also binds towards the end of the Runt domain [59]. *vt34*, *rnt-1(tm388)* and *rnt-1(ok351)* all lack the C-terminal end of the Runt domain, so any *rnt-1* protein made in *vt34* animals has either reduced DNA binding ability or a complete loss of binding. Loss of BRO-1 limits but does not completely abolish the ability of RNT-1 to bind to DNA. *bro-1* mutants exhibit slightly less Swip than *rnt-1* mutants, indicating a reduction in RNT-1 binding is sufficient to induce a significant Swip phenotype.

RUNX transcription factor genes are exquisitely regulated by many different mechanisms including phosphorylation, ubiquitination, acetylation, DNA methylation, and chromatin remodeling [60]. All of these effects involve binding at the TAD or ID domains. In *C*. *elegans*, it has been demonstrated that RNT-1 and BRO-1 both repress *rnt-1* transcription. *rnt-1* expression is high in embryonic stages and decreases in the larval stages, so at the stage we assay Swip, L4, *rnt-1* message levels are relatively low. At this stage, BRO-1 acts to repress *rnt-1* expression, while the TGFβ ortholog DBL-1 activates *rnt-1* expression [61]. In the adult stage, *rnt-1* is expressed in the intestine but it is continuously degraded by the proteasome [62]. The MAP kinase pathway is responsible for stabilizing RNT-1 protein levels in the intestine, in response to oxidative stress. PMK-1 has been shown to phosphorylate RNT-1 *in vitro* [62]. Interestingly, *pmk-1* mutant is slightly more sensitive to 6-OHDA treatment [63–65]. Also, NSY-1 is a MAP3K that acts upstream of PMK-1, mutations in which also provide resistance to the toxicity of not only 6-OHDA but also methamphetamine and MDMA [65].

By qRT-PCR, we observed a significant decrease in mRNA levels of *dat-1* in *vt34* and *rnt-1* mutants. Whether the decrease in mRNA levels results in a similar decrease in DAT-1 protein levels has not been determined, but our 6-OHDA data are consistent with a significant loss of transporters in these mutants. We also observed a decrease in *cat-2* transcript levels. By HPLC, we saw a decrease in DA levels in *dat-1* and *vt34* mutants. Decreased levels of DA could result from loss of DAT-1 mediated DA recycling, decreased *cat-2* mRNA expression or a reduction in CAT-2 activity mediated by presynaptic DOP-2 receptors as a response to increased extracellular DA with loss of DAT-1 protein. It is also possible that there are additional RNT-1 targets that facilitate Swip. Indeed, *dat-1; rnt-1* and *dat-1;vt34* mutants have enhanced Swip compared to N2. These data are consistent with the mutation in *rnt-1* acting in parallel to DA actions via non-dopaminergic cells to support normal swimming behavior. This idea is supported by the opposite shifts we observed in potency of aldicarb and levamisole. Aldicarb sensitivity is typically interpreted as an indicator of the level of ongoing motorneuron ACh release, with the reduced sensitivity we observed interpreted as evidence of reduced ACh release. A reduction in ACh release is consistent with our evidence of increased DA induced inhibition of cholinergic motorneurons. Our observation of increased sensitivity to levamisole, however, indicates that changes have also occurred postsynaptically, specifically an increase in density or activity of ACh receptors, consistent with rnt-1 expression in muscle cells [66]. These findings suggest that coordinate mechanisms supporting both DA signaling to motorneurons and muscle response to motoneurons may be under coordinate control, mediated through RNT-1 dependent mechanisms.

To date, no studies of mammalian RUNX members have identified any relationship between this transcription factor family and DA-dependent phenotypes. Transcripts of *runx2*, required for osteogenesis, as well as *CBFβ* are detected in several different regions of the mouse brain, including the striatum [67]. Immunohistochemistry of RUNX2 has revealed expression in rat cortical astrocytes as well as in a glioma cell line [67]. Given the contribution made by perturbed DA signaling to the pathophysiology of human neuropsychiatric disorders (1–9), studies are warranted to determine if RUNX family members play role(s) similar to *C*. *elegans* RNT-1 in regulating DA signaling.

## Supporting information

S1 Fig Elimination of other candidate mutations recovered in DNA sequencing of the mapped *vt34* genomic region as determinants of Swip. (A) Swip testing of available deletions in gene candidates. Data represents at least 13 trials with data assayed by endpoint manual assays as described in Methods. (B) Expression of *nmtn-1* and *gst-43* does not significantly rescue *vt34* animals. Expression of *pamn-1* does significantly but slightly rescue *vt34* animals. Transgenic animals were assayed for Swip using hand assays. Three transgenic lines per experiment were tested. Data were analyzed using one-way ANOVA with multiple Bonferonni post-tests. ** = *P* <0.01, *** = *P* < 0.001 and error bars represent SEM.
(TIF)

S1 Table Whole genome sequencing mutations unique to *vt34* and *vt31*, within the SNP-mapped region. *Genetic position is based on Worm Base version WS248. **For whole genome sequencing see the Methods section. The WGS mutations in *nmtn-1*, *hasp-1* and *mans-1* were not verified by Sanger sequencing and other nearby mutations were identified instead. ***Based an increase in expression in L4 DA neurons versus whole L4 animals (WormVIZ). # *vt34* animals were injected with a fosmid spanning the indicated gene, or with a PCR product including the genomic locus and at least 1kB of upstream sequence, and. assayed for rescue of the Swip phenotype. More detail is available in the Methods section.
(TIF)
