# Supplementary figures and images for: Dopamine-dependent, swimming-induced paralysis arises as a consequence of loss of function mutations in the RUNX transcription factor RNT-1
Source: PLoS One. 2019 May 13;14(5):e0216417. doi: 10.1371/journal.pone.0216417 (PMC6513266; doi:10.1371/journal.pone.0216417)

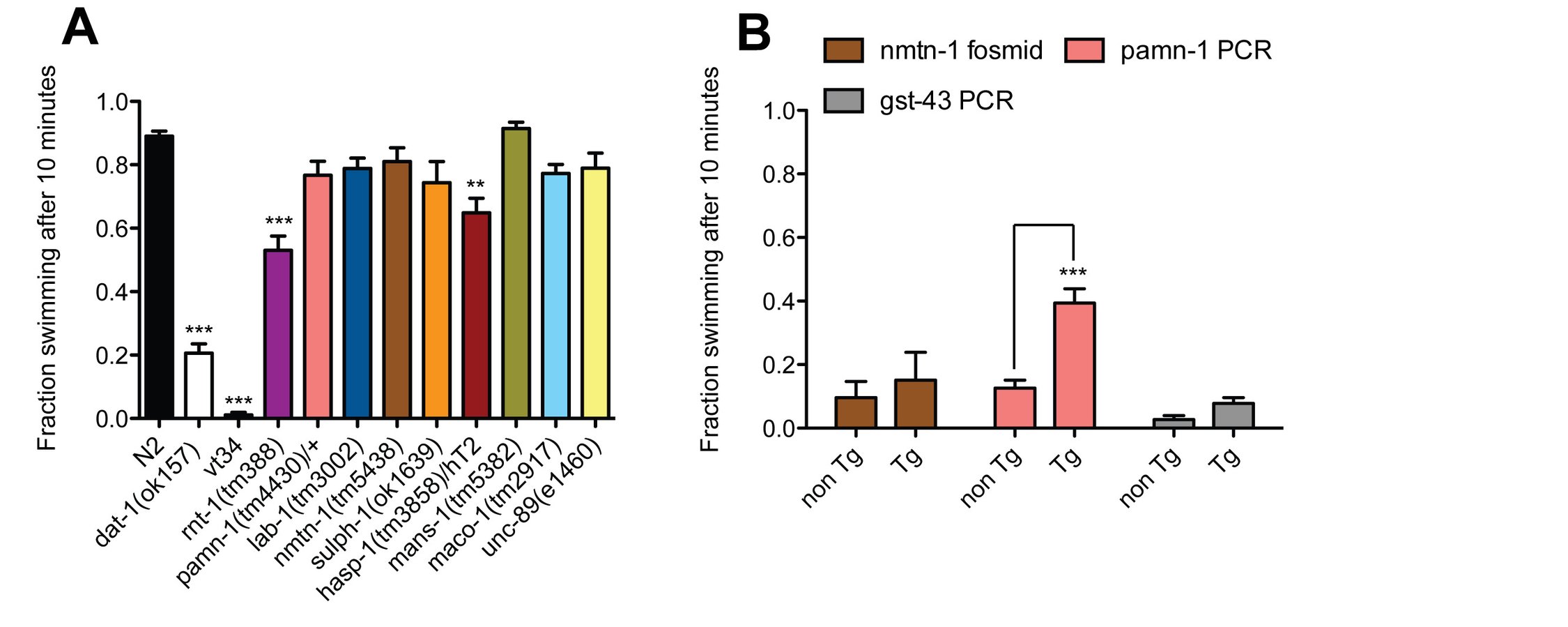

Supplement: S1 Fig — (A) Swip testing of available deletions in gene candidates. Data represents at least 13 trials with data assayed by endpoint manual assays as described in Methods. (B) Expression of nmtn-1 and gst-43 does not significantly rescue vt34 animals. Expression of pamn-1 does significantly but slightly rescue vt34 animals. Transgenic animals were assayed for Swip using hand assays. Three transgenic lines per experiment were tested. Data were analyzed using one-way ANOVA with multiple Bonferonni post-tests. ** = P <0.01, *** = P < 0.001 and error bars represent SEM. (TIF) [file pone.0216417.s001.tif]

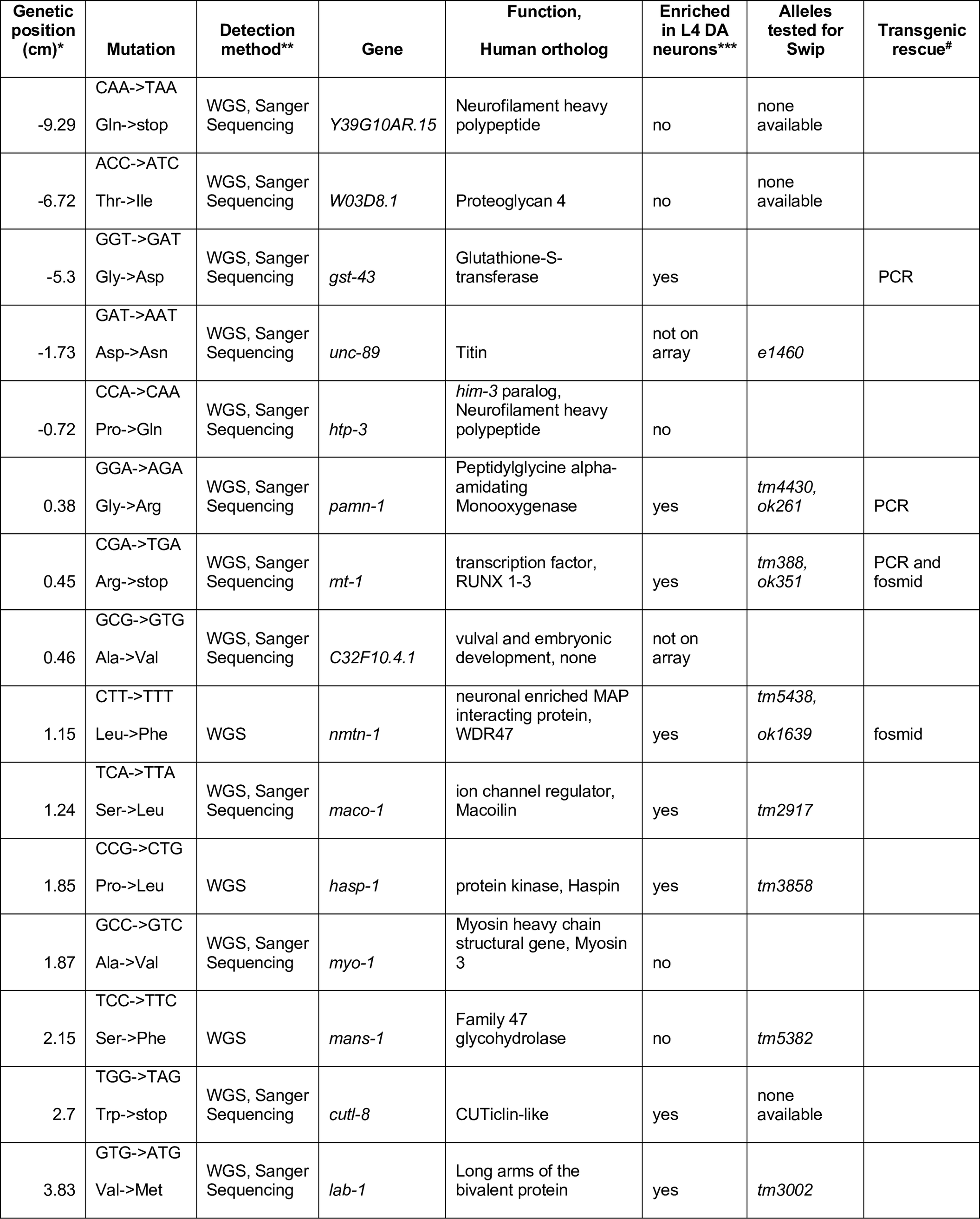

Supplement: S1 Table — *Genetic position is based on Worm Base version WS248. **For whole genome sequencing see the Methods section. The WGS mutations in nmtn-1, hasp-1 and mans-1 were not verified by Sanger sequencing and other nearby mutations were identified instead. ***Based an increase in expression in L4 DA neurons versus whole L4 animals (WormVIZ). # vt34 animals were injected with a fosmid spanning the indicated gene, or with a PCR product including the genomic locus and at least 1kB of upstream sequence, and. assayed for rescue of the Swip phenotype. More detail is available in the Methods section. (TIF) [file pone.0216417.s002.tif]
